# Salient Measures of Hospitalist Workload

**DOI:** 10.1001/jamanetworkopen.2023.28165

**Published:** 2023-08-10

**Authors:** Marisha Burden, Lauren McBeth, Angela Keniston

**Affiliations:** 1 Division of Hospital Medicine, University of Colorado School of Medicine, Aurora

## Abstract

**Importance:**

The ideal hospitalist workload and optimal way to measure it are not well understood.

**Objective:**

To obtain expert consensus on the salient measures of hospitalist workload.

**Design, Setting, and Participants:**

This qualitative study used a 3-round Delphi technique between April 5 and July 13, 2022, involving national experts within and external to the field. Experts included hospitalist clinicians, leaders, and administrators, as well as researchers with expertise in human factors engineering and cognitive load theory.

**Main Outcomes and Measures:**

Three rounds of surveys were conducted, during which participants provided input on the salient measures of hospitalist workload across various domains. In the first round, free-text data collected from the surveys were analyzed using a directed qualitative content approach. In the second and third rounds, participants rated each measure’s relevance on a Likert scale, and consensus was evaluated using the IQR. Percentage agreement was also calculated.

**Results:**

Seventeen individuals from 14 organizations, encompassing clinicians, leaders, administrators, and researchers, participated in 3 rounds of surveys. In round 1, participants provided 135 unique qualitative comments across 10 domains, with 192 unique measures identified. Of the 192 measures presented in the second round, 6 (3%) were considered highly relevant, and 25 (13%) were considered moderately relevant. In round 3, 161 measures not meeting consensus were evaluated, with 25 (16%) considered highly relevant and 95 (59%) considered moderately relevant. Examples of measures considered highly relevant included a patient complexity score and outcome measures such as savings from hospital days avoided and clinician turnover.

**Conclusions and Relevance:**

In this qualitative study measuring hospitalist workload, multiple measures, including those quantifying work demands and the association of those demands with outcomes, were considered relevant for measuring and understanding workloads. The findings suggest that relying on traditional measures, such as productivity-related measures and financial measures, may offer an incomplete understanding of workloads and their association with key outcomes. By embracing a broader range of measures, organizations may be able to better capture the complexity and nuances of hospitalist work demands and their outcomes on clinicians, patients, and organizations.

## Key Points

**Question:**

What are the salient measures of hospitalist workload?

**Findings:**

In this qualitative study with 17 participants, a Delphi technique was used involving national experts to identify and assess measures relevant to hospitalist workload. The measures collected encompassed those that quantified work and reflected the association of workload with workforce and patient quality and safety, as well as financial and productivity-related measures.

**Meaning:**

The findings suggest that incorporating measures that assess both the quantity of work and the association of workload with key outcomes is crucial for evaluating and understanding hospitalist workloads because traditional productivity-focused measures alone may be inadequate.

## Teaser

This qualitative study uses a Delphi technique involving national experts to obtain consensus on the salient measures of hospitalist workload.

## Introduction

High workloads in the hospital setting can have negative associations with patient safety and quality,^1,2,3,4,5,6,7,8^ including delays in care,^8,9,10^ failure to recognize clinical deterioration,^2^ unsafe work conditions,^7^ and preventable adverse events and errors.^11,12,13,14^ Most research on this topic has focused on the nursing field,^2,3,4,15,16^ which resulted in industry standards for nursing staffing ratios and subsequent improvement in workloads. Hospitalist work is characterized by shift work that is high stress and high intensity and involves caring for patients with medically complex conditions. This can lead to unpredictable work hours and schedules, as well as high rates of burnout.^17,18,19^ High workloads can also have negative associations with clinician job performance,^1,8^ clinician health and wellness,^17,20^ and hospital operational^8,9,10^ and financial outcomes.^10,21^ Although the hospitalist care model is widely used in hospitals across the country, the optimal workload and how to measure it are not well understood.^1,22^

Traditionally, the measures for hospitalist workload have focused primarily on starting census (eg, how many patients are on a care team at the beginning of a day), patient encounters, and/or work relative value units (wRVUs).^23^ However, these traditional approaches have limitations because they often only capture the quantity of “things done,” disregarding important factors such as task load,^24^ cognitive load,^25^ and other potential measures, including outcomes associated with high workloads.^1^ The impact of workloads extends beyond productivity-related measures and direct financial considerations. Research increasingly shows the association of workloads with clinician performance, patient safety and quality outcomes, and even organizational performance.^1^ Simply seeing more patients does not necessarily translate into equivalent gains because there may be a productivity paradox characterized by diminishing returns. In other words, the incremental increase in patient volume may not be associated with proportional improvement in outcomes or efficiency.

Studies have shown that 40% of hospitalists stated that their typical patient load in the inpatient setting exceeded safe levels at least monthly^7^ and that resources often are not sufficient for the high workloads.^8^ Given ongoing supply or demand challenges and increasing financial threats, hospitals may be inclined to implement cost-cutting efforts by hiring fewer clinical staff to deliver care to the same or an increasing volume of patients.^1^ The system-level problems that result in increasing workloads can create imbalances between the demands placed on hospitalists and the resources available to them.^26^ These imbalances between job demands and resources are modifiable because they are a result of work structure, processes, and environments.^20^

To establish consensus on the most salient measures of hospitalist workload, we used a 3-round Delphi technique with national experts both within the field and external to the field, including frontline clinicians, administrators, researchers, and leaders. This work represents an initial step toward understanding the measurement of work and work design and its associated outcomes with an aim to eventually develop standards around measurement of hospitalist work.

## Methods

In this qualitative study, we used a 3-round Delphi technique between April 5 and July 13, 2022, to obtain expert consensus of the most salient hospitalist workload measures. We chose this method given the limited literature to date on hospitalist workload measures and to gain opinions from a diverse group of national experts both within the field and external to the field. The Delphi technique is an approach to gather expert opinions on a particular topic through several rounds of surveys,^27,28,29^ with the aim of reaching consensus on findings regarding a specific topic. This technique allows a diverse group of experts from different geographic locations and backgrounds to share expertise and input and to do so anonymously. This study was approved by the Colorado Multiple Institutional Review Board. Participants consented via a postcard consent process. This manuscript adheres to the Standards for Reporting Qualitative Research (SRQR) reporting guideline^30^ and the Guidance on Conducting and Reporting Delphi Studies (CREDES).^29^

### Expert Panel and Sampling Strategy

National experts from both within the field of hospital medicine and external to the field were invited to participate. Experts were recruited based on their (1) experience delivering care to hospitalized patients as frontline hospitalists, inclusive of physicians and advanced practice clinicians; (2) experience as a health care executive, business administrator, or leader; (3) expertise in health care quality and safety; and/or (4) expertise in human factors engineering and cognitive load theory. Our aim was to include a range of participant expertise,^31^ including clinicians, leaders, administrators, researchers, and experts from fields within and outside of medicine who possess additional expertise relevant to the research question. We also felt that having administrative leaders (physicians and otherwise) was important because this group often plays an active role in assessing workloads and allocating resources based on those measurements. Potential participants each received invitations to participate by email and were emailed no more than 3 times. Participants were offered a stipend of $200 if they agreed to participate. The identity of the participants was not shared with the other participants.

### Description of the Delphi Technique

The Delphi technique involved 3 rounds of surveying the expert participants. In the first round, the participants were asked open-ended questions about potential workload measures and were prompted to think about these measures under the following domains: productivity; financial; workload; hospitalist-related or worker-related measures; quality and safety; job performance; institutional measures; academic measures; diversity, equity, and inclusion measures; and “other” (see the eFigure in Supplement 1 for the survey). The domains used in this study were based on a review of the literature and the previous research experience of the study team. To ensure an unbiased exploration of workload measures, we deliberately refrained from providing specific definitions for the domains. The qualitative data collected from the first round were analyzed using a directed qualitative content approach to distill hospitalist workload measures from the unstructured comment data.^32^ Measures that were repeated across participants were aggregated into a single measure to reduce redundancy. All suggested measures were included in round 2.

For the second round, participants were provided with anonymous aggregated data of all measures identified from round 1. Participants were asked to rate each measure on a 0 to 7 Likert scale of relevance to hospitalist work. Ratings of 0 to 2 were considered not relevant, 3 to 5 were considered moderately relevant, and 6 or 7 were considered very relevant. Consensus across individuals within the second and third rounds was evaluated using the IQR calculated for the median value for each item ranked.^28,33^ If the IQR was 1 or below on the 0 to 7 Likert scale, consensus was considered obtained. The percentage of participants who considered a measure moderately or highly relevant (ie, percentage agreement) was also calculated for each item after each round to evaluate the level of agreement between individuals. A percentage of 75% or more indicated that respondents had reached agreement.

In the third round, the participants received quantitative group results (median, minimum, and maximum score for each measure) as well as aggregated qualitative feedback. They also received individual reports that allowed participants to compare their responses with the overall group results. They were then able to rerate their responses using the same methods as in round 2. This round was limited to measures about which the participants did not find consensus in the second Delphi round, meaning the IQR around the median value exceeded 1.

To enhance trustworthiness, the preliminary findings were shared via email with 5 workforce experts, including physicians, health systems experts, and administrative leaders, to seek feedback and validation of the results, asking if the results seemed valid based on their lived experiences and if they saw any issues or concerns with the findings.^29,30^ Of the 5 experts we emailed, feedback was received from 4. The feedback confirmed that the findings seemed valid and the comments informed some of the discussion points.

### Statistical Analysis

Analyses were performed using SAS Enterprise Guide, version 8.3 (SAS Institute Inc) and Microsoft Excel for Microsoft 365 MSO (version 2306, build 16.0.16529.20100) 64-bit (Microsoft Corp).

## Results

We used a 3-round Delphi technique between April 5 and July 13, 2022, with 17 individuals from 14 organizations, including clinicians, researchers, and individuals in leadership roles. Twenty-three individuals were initially approached to participate, with 17 individuals participating in the study (74% participation rate). Participants from geographically diverse organizations were included, with 3 of the organizations having 2 participants and the remaining each having 1. Most participants were from academic institutions (n = 15). Participants included physicians and physician leaders (13 of 17 [76%]); 3 individuals with PhDs (18%) with a variety of roles, including a health services researcher, research psychologist with expertise in human factors engineering and cognitive load, and a health care operations manager; and 1 advanced practice clinician (6%). See Table 1 for the demographic characteristics of the participants and the Figure for the Delphi flowchart. In each of the 3 rounds, we had between 76% (13 of 17) and 88% (15 of 17) participation.

**Table 1.** Demographic Characteristics of the Delphi Panel

Characteristic	No./total No. (%)
Gender	
Women	7/17 (41)
Men	10/17 (59)
Occupation	
Physician	13/17 (76)
Chief executive officer	1/17 (6)
Chief medical officer	1/17 (6)
Director, division of hospital medicine	5/17 (29)
Executive director	1/17 (6)
Advanced practice clinician	1/17 (6)
PhD	3/17 (18)
Healthcare operations management	1/17 (6)
Health services researcher	1/17 (6)
Research psychologist	1/17 (6)
Geographic region of organizations	
Northeast	4/14 (29)
Midwest	3/14 (21)
South	2/14 (14)
Southwest	1/14 (7)
West	4/14 (29)

**Figure.**
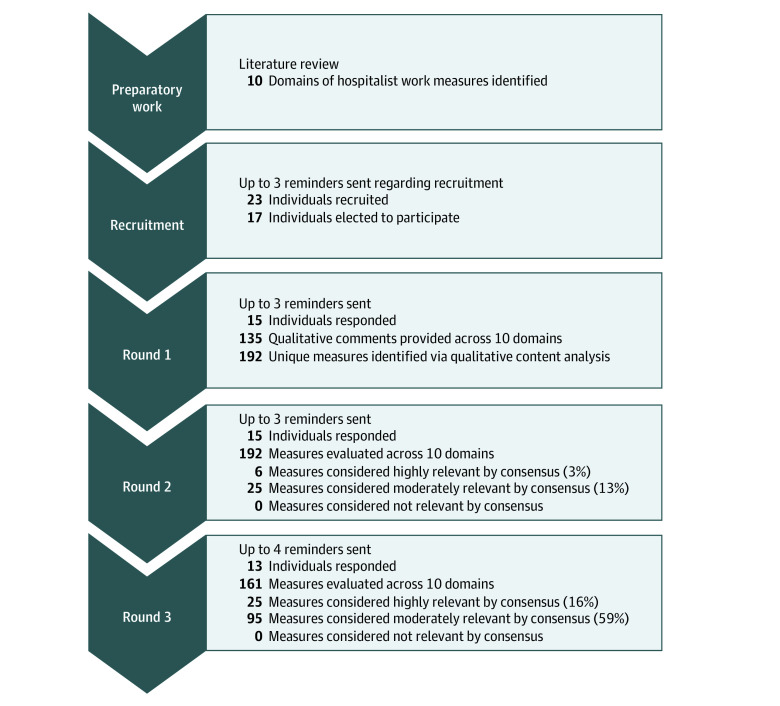
Delphi Flowchart

In round 1, we received 135 unique qualitative comments provided across 10 domains, and 192 unique measures were identified (Box). Of the 192 measures presented in the second round, 6 (3%) were considered highly relevant by consensus, and 25 (13%) were considered moderately relevant by consensus (Table 2). In round 3, the 161 remaining measures not meeting consensus were reevaluated across the 10 domains, with 25 measures (16%) considered highly relevant by consensus and 95 measures (59%) considered moderately relevant by consensus. The measures considered highly relevant included a patient complexity score, the Maslach Burnout Inventory, wRVUs, savings from hospital days avoided, and patient clinical severity (Table 3). Measures included those that evaluated workloads as well as measures that were outcomes that may be associated with workloads. In addition, modifiers were also thought to be relevant: modifiers such as type of work (eg, teaching service vs direct care, or clinical severity) may be associated with the workload measures. The full list of measures for each round, the IQR, and the assessment of relevance, including those that did not meet consensus criteria, are provided in eTables 1 and 2 in Supplement 1. Measures that did not reach consensus fell across multiple domains. Examples included discharge before noon, percentage early discharge, collections, subsidy per full-time equivalent, downstream clinical revenue from having hospitalists, time spent in the electronic health record, institutional measures such as pressure injuries, Hospital Consumer Assessment of Healthcare Providers and Systems Measures, and *US News & World Report* rankings.

Box. **Qualitative Findings of Round 1**

**Exemplar Quotes for Productivity Measures Domain**

Productivity measures should encompass all the different missions that hospitalists serve—and not just the clinical one. (Hospitalists are clinicians, scholars, mentors, advocates, QI experts, administrators.) For the clinical mission it should be the numbers of patient contacts (rather than RVUs) (respondent 3).
Care should be taken to not incentivize having a high census, as this could inadvertently disincentivize discharging patients. Hospitalists are usually not responsible for how many patients are on their service—it is determined by who is sick and needs hospital admission (respondent 6).
Should not use independent method of only looking at starting patient volumes, amount billed, and amount collected because this does not assess efficiency. If we could look at productivity from a standpoint of reducing length of stay and readmissions and improving the turnover of beds (another possible metric) then we could actually see more patients per bed than if we solely measure productivity based on increasing starting censuses and patient volumes (respondent 9).
In general, it is preferable to have a mix of structure, process and outcome measures (which hopefully have existing established relationships to each other). I always want to cite Donabedian’s conclusions that there are no single best measures of quality and judgement must be based on multiple measures across key areas (like judiciously selected geological borings) (respondent 13).

**Exemplar Quotes for Financial Measures Domain**

Baseline salaries should be based on training (eg, more if also palliative care fellowship trained) plus years since completion of training and should not differ otherwise. Bonus structure should be linked to organizational goals: additional clinical work, evidence of outstanding quality, etc (respondent 6).
Financial measures can be related to patient outcomes to promote higher quality of care. It can be based on patients’ satisfaction, average length of stay, number of readmissions, mortalities, etc. Financial measures should also account for hospital location, size, hospitalist seniority, etc (respondent 12).

**Exemplar Quote for Workload Measures Domain**

I have been wondering about populations that need more time from us to provide care that they can trust. While identifying populations seems like another type of stereotyping—perhaps we should start by assuming that some patients deserve more time and therefore should be counted twice for our census. Patients who are labeled “difficult,” those who need an interpreter, those who need goals of care conversations, etc—meaning—we need to find a way to apportion more time where it is necessary for great care. We could estimate the prevalence of such patients in each population and adjust our expected patient load accordingly (respondent 3).

**Exemplar Quote for Hospitalist- or Worker-Related Measures Domain**

While assessments of burnout are widely used, I cannot help but think that the best assessment of the hospitalist’s well-being may be the answer to the question “Did you feel like you provided the best care you could today?” (respondent 3)

**Exemplar Quotes for Quality and Safety Measures Domain**

I could go on for a bit here—but I think the crux of it is that we should measure things that are within the locus of control for each individual hospitalist (respondent 3).
Quantitative measures for individual hospitalist performance will be very difficult to determine in a valid fashion. So much of clinical outcomes is (a) related to patient characteristics and (b) the results of several clinicians working overtime and day and night. Attribution of outcomes, quality, and safety of patient care is a central thorny issue. Suggest instead process measures such as implementing delirium precautions, consistent medication reconciliation, rates of follow-up appointments provided with discharge paperwork, etc (respondent 6).
Important that any and all of these are evaluated with a team approach so that we can advocate for appropriate resources. I think looking at quality measures like inpatient mortality, LOS, and readmissions is only helpful if done in “aggregate” with CM and SW, PT and OT, and system-level resources such as ability for follow-up and outpatient treatment (respondent 9).
Outcomes data analysis is tough given hard-to-attribute CAUTI, CLABSI, falls, etc, rates to one specific group or person. HCAHPS are unreliable (and often inaccurate) but important to understand (respondent 11).
Overworking can cause burnout and create an unsafe environment (respondent 12).

**Exemplar Quote for Job Performance Measures Domain**

Depending on the hospitalist’s niche—more emphasis on the identity that they value—meaning—if you want to be an educator—then something about teaching evaluations (respondent 3).

**Exemplar Quotes for Institutional Measures Domain**

However, it would be important to know the workflow process across the different staff involved, which probably varies greatly by facility (respondent 13).
Groups (rather than individuals) could be measured and compared based on burnout scores, turnover; this might lead to industry changes based on factors identified within groups that lead to less burnout and turnover (respondent 4).

**Exemplar Quote for Academic Measures Domain**

The ability to collaborate and involve others—success measured by inclusion, not exclusivity (respondent 3).

**Exemplar Quotes for Diversity, Equity, and Inclusion Measures Domain**

Pay parity between genders and FTE apportioning for nonclinical roles—women seem to get smaller FTEs for nonclinical roles than men do. Racial and ethnic makeup of the hospitalist group—and how concordant it is with the population that group serves. Racial and ethnic makeup of the hospitalist group leadership. Presence of objective criteria for positions and awards. Availability of in-person translators for different languages and sign languages. The hospitalist group should be monitoring patient outcomes stratified by race, ethnicity, and English proficiency (respondent 3).
Diversity, equity, and inclusion measures are really about the organization, not the individual. For the organization: diversity among leadership teams measured in a standard fashion accounting for race, ethnicity, gender, sexual identity, age, etc. Number and diversity of applicants for open calls for leadership positions. Compensation measures, adjusted for seniority, academic level, but aimed at making sure equity across the division. Inclusion: composition of committees, task forces, initiatives measured through a diversity lens. Survey data assessing perception of equity and inclusion across the division (respondent 8).

**Exemplar Quotes for Other Measures Domain**

At the heart of it, we are here to take care of patients. Yet, there is no description of what constitutes an excellent hospitalist. Some of what I put down in quality and safety would go into it—but there is more—like communication skills, the quest for lifelong learning, etc. And I would love to see something that would help us recognize, reward, and replicate excellence! (respondent 3)
Also, how and when you measure may be as important as what to measure. To that end, I would suggest the following: include a variety of hospital types, sizes, and locations; try to randomly select dates and times (over 24 hours) for measurement consistently across sites, ideally with variation across months in case of seasonality. Also, it would be helpful to capture differences in workflow (algorithms) across sites to understand local context in relation to staffing levels for others involved in the workflow (respondent 13).

Abbreviations: CAUTI, catheter-associated urinary tract infection; CLABSI, central line–associated blood stream infection; CM, care management; FTE, full-time equivalent; HCAHPS, Hospital Consumer Assessment of Healthcare Providers and Systems; LOS, length of stay; OT, occupational therapy; PT, physical therapy; QI, quality improvement; RVU, relative value unit; SW, social work.

**Table 2.** Quantitative Results of Round 2: Measures Considered Highly Relevant by Consensus

Domain	Measure	Median (IQR)	Agreement, No./total No. (%) (N = 15)
Scoring systems	Patient complexity score: social, behavioral, language barriers, medical acuity	6.5 (1)	14/15 (93)
Well-being and culture	Maslach Burnout Inventory	6 (1)	15/15 (100)
Productivity	Work relative value units	6 (1)	13/15 (87)
Financial measures	Savings from hospital days avoided	6 (1)	13/15 (87)
Modifiers^a^	Clinical severity	7 (1)	14/15 (93)
Modifiers^a^	Observed to expected ratios for measures, which assess the deviation from expected values	6 (1)	12/15 (80)

^a^ The domain of “Modifiers” was chosen when measures were observed to have a broad association with outcomes across multiple domain areas.

**Table 3.** Quantitative Results of Round 3: Measures Considered Highly Relevant by Consensus

Domain and measure	Median (IQR)	Agreement, No./total No. (%) (N = 13)^a^
Scoring systems		
Overall score: scoring system that includes length of stay, total visits, mean discharge time, patient complexity, work relative value units	6 (1)	13/13 (100)
Well-being and culture		
Turnover, intent to leave	7 (1)	12/12 (100)
Engagement	6 (0.5)	12/12 (100)
Productivity		
Encounters: admissions, follow-ups, discharges, consultations	6 (0)	13/13 (100)
Shifts worked	6 (0)	13/13 (100)
Mean starting census	6 (0)	13/13 (100)
Follow-up visit ratio: number of discharges and follow-up encounters for patients still hospitalized for a given period of time expressed as a percentage	6 (1)	13/13 (100)
Financial		
Cost of care	6 (1)	11/11 (100)
Savings from readmissions avoided	6 (1)	12/13 (92)
Intensive care unit days avoided	6 (1)	12/13 (92)
Workload		
“Pajama time”: hours spent in the electronic health record from home or outside scheduled shift	6 (1)	13/13 (100)
Rates of multitasking	6 (1)	12/13 (92)
Pages, calls, and messages per shift: number and time	6 (1)	13/13 (100)
Time spent on discharge planning	6 (1)	13/13 (100)
Time spent on clinical decision-making	6 (1)	13/13 (100)
Patient acuity	6 (1)	13/13 (100)
Measure of patient intensity of care: number of orders, number of procedures, geographical location	6 (1)	13/13 (100)
Hospitalist to patient ratio	6 (1)	13/13 (100)
Quality and safety		
Diagnostic error rate	6 (1)	12/13 (92)
Job performance		
How likely would you refer a family member for care by this clinician?	6 (1)	13/13 (100)
Modifiers^b^		
Unit of time: per shift, per day, per year	6 (1)	11/11 (100)
Unit of clinician: team, physician, advanced practice clinician	6 (1)	11/11 (100)
Unit of effort: cFTE, total effort	6 (0)	10/10 (100)
Type of work: direct care, teaching, comanagement	6 (1)	12/12 (100)
Per work relative value unit	6 (1)	10/10 (100)

Abbreviation: cFTE, clinical full-time equivalent.

^a^ Missing responses were not included in denominator.

^b^ The domain of “Modifiers” was chosen when measures were observed to have a broad association with outcomes across multiple domain areas.

## Discussion

In this study, we used a well-accepted consensus technique to gather expert opinion on the most important measures of hospitalist workload. The measures collected encompassed those that quantified work, reflected the association of workload with workforce and patient quality and safety, and included financial and productivity-related measures. Our findings suggest that traditional measures of hospitalist workload, which often focus solely on productivity, do not provide a comprehensive assessment of workload.

Traditional measures for productivity in the hospitalist field (as well as most other specialties) revolve around counting specific tasks or activities performed, such as patient encounters, patient census, or wRVUs.^23^ As expected, these types of measures were found to be relevant by the participants in this study. However, the measurement of workload in this study extended beyond those quantitative measures, including a wide array of measures that captured workloads but also acknowledged the link to other key outcomes. This study highlights the need to consider various approaches to measuring workload and understanding its overall association with outcomes. This parallels the notion of how productivity and compensation are often linked in practice; however, the same level of attention and connection is rarely present in regard to quality outcomes or clinician-related outcomes. This finding suggests a crucial area of future study.

The concept of modifiers was an important finding in this work. The domain of modifiers was used to describe measures that had a broad association with outcomes across multiple domains. For instance, when assessing the number of patients seen, it is important to consider clinical severity. Similarly, it is also important to consider the observed to expected ratios for measures, which assess the deviation from expected values that are adjusted for factors such as acuity of illness.

Our work builds on previous work that assessed characteristics associated with attending physician workloads^34^ and incorporated measures beyond those typical of productivity and financial models. The current study findings align with a previously published framework,^1^ which uses a total worker health approach^35^ for designing and implementing work structure and processes that support the workforce to deliver high-quality care while also benefiting institutional and worker outcomes. This framework also aligns with the Donabedian principles,^36^ which recognize the interplay between structure, process, and outcomes in health care.^37^

In our study, we found that consensus was reached on 31 highly relevant measures and 120 moderately relevant measures, leaving 41 measures without consensus. There were zero measures considered not at all relevant by consensus. Some of the measures may be difficult to capture electronically, while others are already readily available at most institutions. Although our study provides a list of potential measures for assessing hospitalist workload, more research is needed to determine how these measures (and which measures) can be effectively used in practice to address optimal workloads and improve patient care, recognizing that a much smaller list will likely be needed.

Developing an understanding of how hospitalist workloads impact job performance, the workforce, patient safety and quality, and other organizational outcomes is paramount. The results of this study set the stage for future research on hospitalist workloads and provide a starting point for discussions around interventions to measure and optimize workloads. Future work could be used to inform policy decisions related to workforce development and retention, such as workload strategies for reducing burnout and promoting well-being among hospitalists. Furthermore, future work should investigate how different workload-sharing strategies, such as collaboration with advanced practice clinicians or learners, can be measured and evaluated. This work nicely pairs with emerging areas of innovation that include using electronic health record use measures (ie, electronic health record audit log data) to measure and monitor workloads and work patterns.^38,39,40,41^ Activity in this area of research has been predominantly in the outpatient setting; however, it represents an area that should be focused on in the inpatient setting, in particular when paired with outcomes.

Many qualitative comments highlighted the importance of acknowledging the total work effort of hospitalists, including clinical as well as nonclinical effort. For example, hospitalists often serve as quality experts for their institutions, but this time and effort may not be compensated. Another example is the work effort involved in coordinating transfer center calls, which generate additional business for the organization but typically do not directly generate wRVUs for the clinician providing the transfer call coordination services. Similarly, it is important to acknowledge the work effort involved in academic endeavors, such as teaching and scholarly work, because these are associated with total work effort. The topic of how the work of physicians in partnership with advanced practice clinicians is captured did not come up. Since the completion of this study, there have been changes in the Centers for Medicare & Medicaid Services billing rules.^42^ As a result, although physicians may contribute to patient care oversight and decision-making, their efforts may not be captured in measures such as wRVUs or encounters depending on which clinician provides the substantive portion of the visit, which may be associated with challenges in capturing work. Last, while there are many measures to consider, perhaps it may be as simple as 1 participant proposed: “Did you feel like you provided the best care you could today?”

### Strengths and Limitations

Our study has several strengths, including representation from a variety of fields, diversity in geographic location, and high participation rates in each round. Thus, the findings represent broad views, including those of clinicians, those in charge of financial decision-making, and experts from fields both within and external to medicine.

Our study also has some limitations. Although this diverse representation adds value, there may have also been inherent biases. We did not assess the percentage of clinical effort of participants, which could have influenced responses. Leaders, including nonclinicians, were also part of the study, and while their involvement in understanding the day-to-day work may vary, their perspectives can contribute to insights from a broader organizational view and research standpoint. In addition, the Delphi technique was used to gather expert opinions on measures of hospitalist workload; these opinions do not necessarily imply the correct answer or judgment.^29^ The number of measures considered highly relevant was still a rather large number; thus, additional work to understand which measures are most critical will be important next steps. In addition, most experts represented individuals from academic settings, and the Delphi technique was conducted with predefined rounds, which may have led to the premature exclusion of certain measures.

## Conclusion

In this qualitative study of workload measurement, multiple measures, including those quantifying work demands and the association of those demands with outcomes, were considered relevant for measuring and understanding hospitalist workloads. The findings suggest that solely relying on conventional measures, such as productivity-related measures and financial measures, may provide an incomplete understanding of hospitalist workloads and their association with important outcomes.

## Notes

Supplement 1. **eFigure.** Survey Instrument **eTable 1.** Delphi Panel Round 2 Results **eTable 2.** Delphi Panel Round 3 Results

Supplement 2.
Data Sharing Statement
