# Supplementary material for: Salient Measures of Hospitalist Workload
Source: JAMA Netw Open. 2023 Aug 10;6(8):e2328165. doi: 10.1001/jamanetworkopen.2023.28165 (PMC10415953; doi:10.1001/jamanetworkopen.2023.28165)
Supplement: Supplement 1. — eFigure. Survey Instrument eTable 1. Delphi Panel Round 2 Results eTable 2. Delphi Panel Round 3 Results [file jamanetwopen-e2328165-s001.pdf]

Supplementary Online Content

Burden M, McBeth L, Keniston A. Salient measures of hospitalist workload. *JAMA Netw Open.* 2023;6(8):e2328165. doi:10.1001/jamanetworkopen.2023.28165

eFigure. Survey Instrument

eTable 1. Delphi Panel Round 2 Results

eTable 2. Delphi Panel Round 3 Results

This supplementary material has been provided by the authors to give readers additional information about their work.

© 2023 Burden M et al. *JAMA Network Open.*

eFigure. Survey Instrument

Measuring Hospitalist Work - Delphi Panel Survey 1

Thank you for agreeing to participate in this important project! Instructions: Please enter as many measures as possible for the following categories on ways to measure hospitalist work. Feel free to think big–even if it isn't currently possible to measure – what should we measure? We anticipate this will take approximately 15 minutes to complete. Your responses will be confidential. You can return to the survey if you are not able to complete everything in one sitting.

Please note that your email and name will be stored separately from your responses to the questionnaires and your identity and respective input will not be shared with the other panelists.

Name: ____________________________

Preferred email for Delphi Panel communications: ____________________________

Productivity measures:

Financial measures:

Workload measures:

Hospitalist/worker related measures:

Quality and safety measures:

Job performance measures:

Institutional measures:

Academic measures:

Diversity, equity, and inclusion measures:

Other measures:

© 2023 Burden M et al. *JAMA Network Open.*

eTable 1. Delphi Panel Round 2 Results

Measure	Median	IQR	Consensus	% Agreement Moderately or Very Relevant
Patient complexity score: social, behavioral, language barriers, medical acuity	6.5	1	Yes	93%
Overall score: scoring system that includes length of stay, total visits, average dc time, patient complexity, work relative value units	5.5	2	No	87%
Clinical documentation query response rates	4	3	No	67%
Appropriate billing codes; including for prolonged visits, critical care time, counseling, coordinating care	4	3	No	80%
Major complication or comorbidity capture rates	5	3	No	87%
Diagnosis related groups	4	3	No	87%
Turnover, intent to leave	6	2	No	100%
Maslach Burnout Inventory	6	1	Yes	100%
Engagement	6	2	No	100%
Work-life satisfaction	6	2	No	100%
Discharges before noon	3	2	No	73%
Percent early discharge	3	2	No	67%
Length of stay	4	2	No	80%
Bed turnover	4	2	No	80%
Time to procedure: from order	3	2	No	80%
Time spent on transfer center calls	3.5	3	No	67%
Encounters: admissions, follow ups, discharges, consults	6	2	No	93%
Encounters: critical care time, procedures	5	2	No	93%
Work relative value units	6	1	Yes	87%
Shifts worked	6	2	No	93%
Average starting census	6	2	No	93%
Follow-up visit ratio: number of discharges/follow-up encounters for patients still hospitalized for a given period of time expressed as a percentage	5	2	No	87%
Collections	4	2.5	No	60%
Subsidy per Full-time equivalent (FTE)	3	3	No	53%
Professional fee charges/collections: per admission/per hospitalization	4.5	2.5	No	60%
Percent of division revenue from professional billing	4	3	No	47%
Percent working minutes billed per day	2	3	No	33%
Financial support from hospital	4	3	No	60%
Revenue beyond clinical work	4	2	No	73%
Downstream clinical income from having hospitalists	5	2	No	87%

© 2023 Burden M et al. *JAMA Network Open.*

Dollar stream from outside hospital calls	5	2.5	No	67%
Cost of care	6	3	No	73%
Savings from readmissions avoided	6	2	No	87%
Savings from hospital days avoided	6	1	Yes	87%
Intensive care unit days avoided	6	3	No	87%
NASA Task Load Index	5	1	Yes	80%
Hours per type of shift	5	1	Yes	93%
"Pajama Time": hours spent in the electronic health record from home or outside scheduled shift	6	2	No	100%
Average number of interruptions experienced by a hospitalist while working directly with a patient/family	5	3	No	100%
Number of critical events or emergencies the hospitalist has to respond to within a week	5	1	Yes	100%
Number of times within a week or month that essential tools or supplies needed in working with patient are not readily available	5	1	Yes	100%
Rates of multitasking	6	2	No	33%
Number of orders placed: electronically or verbally	4	2	No	100%
Coordination time: complexity of the patient by numbers of consultants involved	6	3	No	100%
Pages/calls/messages per shift: Number and time	5	2	No	93%
Time spent on discharge planning	6	2	No	100%
Time spent in the electronic health record: including breakdown on communication in electronic health record, documentation	6	2	No	100%
Time spent on clinical decision making	6	2	No	93%
Patient acuity	6	2	No	93%
Non-relative value unit generating work: outside hospital transfer calls, peer to peer	5	2	No	100%
Number of interactions with other care team members	5	2	No	87%
Measure of patient intensity of care: number of orders, number of procedures, geographical location	7	2	No	100%
Fraction of workday devoted for different tasks such as teaching, working with electronic records	5	1	Yes	100%
Hospitalist to patient ratio	6	2	No	100%
Administrative time	5	1	Yes	87%
Meeting volume	5	1	Yes	73%
Presentation prep and performance time	5	2	No	87%
Self-assessment of quality of care	5	3	No	87%
Number of additional shifts worked beyond scheduled shifts	5	2	No	100%
Quality of communication	5	3	No	87%
Number of consults called	5	2	No	93%
Jeopardy or paid time off used	4	2	No	80%
Number of days worked in row	5	1	Yes	100%

© 2023 Burden M et al. *JAMA Network Open.*

Number of overtime hours	5	1	Yes	100%
Number of hospitalists by rank	5	4	No	80%
Number of hospitalists by position	5	2	No	87%
Number of hospitalists by seniority	6	2	No	87%
Number of hospitalists by teaching status	5	3	No	93%
Number of hospitalists by research status	5	5	No	67%
Hospitalist demographics: age, gender, years with division	5	2	No	93%
Rates of offer acceptance	4.5	2	No	73%
Medication safety: high risk meds, error rate	5	4	No	87%
Foley management	3	3	No	73%
Rate of readmission	5	5	No	73%
Rate of inhospital mortality, observed to expected	5	4	No	80%
Antibiotic usage	4	2	No	87%
Number or rate of appropriate transfers	4.5	3	No	73%
Number of patients discharged before medically ready per month	5	3	No	53%
Appropriate transfusion thresholds	4	1	Yes	80%
Rates of hypoglycemia	4	2	No	80%
Rates of contradictory medical cocktails	4.5	3	No	73%
Documentation of reason for dual antiplatelets or anticoagulant	5	2	No	80%
Rates of appropriate venous thromboembolism prophylaxis	5	2	No	87%
Participation and/or leadership of quality working group or project	5	1	Yes	93%
Participation in morbidity/mortality conferences	5	1	Yes	87%
Participation in quality/safety committees	5	3	No	87%
Number of "early identification of deterioration"	4	1	Yes	80%
Delirium precautions initiated	4	2	No	73%
Medication reconcilliation	5	4	No	80%
Follow-up appointments made at discharge	5	4	No	67%
Diagnostic error rate	6	3	No	87%
Percent warm handoffs to primary care provider	4	3	No	80%
Percent irrelvant studies	5	1	Yes	73%
Number of excess daily labs	5	3	No	93%
Number of excess daily clinical procedures	5	3	No	73%
Percent of patients with addressed goals of care	4	3	No	93%
Percent of patients who are non-English speaking for whom interpretation is used	5	2	No	93%
Group education initiatives: ex., in-hospital and discharge opiate utilization	5	2	No	87%

© 2023 Burden M et al. *JAMA Network Open.*

Best practice order set utilization	4	2	No	73%
Goal-directed therapy utilization	4.5	2	No	73%
Agency for Healthcare Research and Quality (AHRQ) Hospital Survey on Patient Safety Culture	4	4	No	73%
Rapid response team or code blue calls	5	3	No	87%
Intensive care unit readmission rate	4	3	No	87%
Rate of unanticipated transfer from floor to intensive care unit	5	3	No	87%
Peer feedback on quality of care	5	3	No	93%
Discharge summary completion rate	6	3	No	87%
Clinical documentation completeness	5	2	No	93%
Communication and collaboration	6	2	No	93%
Meeting attendance	5	3	No	80%
Overuse of diagnostic tests or procedures	5	4	No	87%
Quality of relationships with peers and trainees	5	3	No	93%
Peer evaluations	5	1	Yes	93%
Trainee evaluations	5	2	No	87%
Involvement with quality improvement efforts	5	1	Yes	93%
Quality of handoffs or signouts	6	3	No	93%
Responsiveness to feedback	5	4	No	93%
How likely would you refer a family member for care by this clinician?	6	2	No	87%
Assessment of professionalism	6	3	No	80%
360 degree evaluations	5.5	2	No	80%
Academic output	5	4	No	67%
Presentations, publications, or committee participation	4.5	3	No	73%
Participation in job-related activities	5	1.5	No	73%
Meeting institutional requirements for advancement or promotion	4.5	4	No	67%
Committee participation	5	2	No	73%
Leadership roles	4.5	3	No	73%
Dissemination of academic work through other venues	4	2	No	73%
Pressure injuries	4	4	No	60%
Central line-associated bloodstream infections	5	4	No	60%
Catheter-associated urinary tract infections	4	4	No	60%
Clinical documentation integrity	5.5	3	No	73%
Hospital Consumer Assessment of Healthcare Providers and Systems measures	2	3	No	40%
Amount of funding provided for academic pursuits	4	3	No	60%
Grant funding	2	2	No	40%

© 2023 Burden M et al. *JAMA Network Open.*

US News & World Report ranking	2.5	4	No	47%
Doximity ranking	2	4.5	No	33%
Volume/growth of system	4.5	2.5	No	67%
Clostridium difficile infections	4	3	No	67%
Centers for Medicare & Medicaid Services quality measures	5	4	No	73%
Number of trainees mentored or supervised per month	5	2	No	87%
Time spent with trainees in hours	5	3	No	73%
Teaching time	5	2	No	73%
Grand rounds given	4	1	Yes	73%
Rates of promotion	4	4	No	60%
Teaching evaluations	5	3	No	67%
Number of abstracts accepted	4	2	No	67%
Impact score	3.5	3.5	No	47%
Grants submitted	3	3	No	47%
Grants funded	4	4	No	47%
Total grant funding	4	4	No	47%
Medical education leadership positions	4	2	No	73%
Hours of pre-clinical medical student teaching	3.5	2	No	67%
Number of journal articles authored	3	3	No	60%
Number of journal articles published	3	4	No	60%
Hours of additional training or continual education per year	4	3	No	67%
Lectures or talks given	4	1	Yes	67%
Letters of recommendation written	4	2	No	60%
Resident conferences attended	4	1	Yes	67%
Curriculum developed or implemented	4	1	Yes	73%
Courses directed	4	0	Yes	73%
Interviews with students and residents	4	2	No	67%
Clinical Competency Committee participation	4	2	No	53%
Student preceptor hours	3.5	2	No	53%
Professional organization involvement	3.5	2	No	73%
Mentorship hours	4	1	Yes	73%
Publication reviewer work	3	2	No	60%
Hours spent in educational activities or preparation	4	2	No	80%
Amount of teaching service by gender, race, ethnicity, language abilities	5	2	No	87%
Academic rank and promotion by gender, race, ethnicity, language abilities	5	4	No	80%

© 2023 Burden M et al. *JAMA Network Open.*

Leadership positions by gender, race, ethnicity, language abilities	5	2	No	87%
Committee membership by gender, race, ethnicity, language abilities	5	1	Yes	87%
Hiring by gender, race, ethnicity, language abilities	5	3	No	87%
Applicant interviews by gender, race, ethnicity, language abilities	5	2	No	87%
Percent of patients with limited English proficiency getting interpreter services	5	2	No	80%
Pay parity by gender, race, ethnicity	6.5	2	No	87%
FTE apportioning for non clinical roles by gender, race, ethnicity	6	3	No	80%
Concordance of race/ethnicity of hospitalist group with patients receiving care	3.5	2	No	73%
Perceptions of diversity, equity, and inclusion across division	5	4	No	73%
Completion of institutional diversity, equity, inclusion (DEI) training	5	3	No	73%
Participation in diversity, equity, and inclusion work	4	2	No	73%
Use of stigmatizing language in the medical record	4	3	No	73%
Clinical severity	7	1	Yes	93%
Geographic spread	5	3	No	93%
With or without advanced practice provider	5	2	No	100%
Unit of time: per shift, per day, per year	5.5	2	No	87%
Unit of provider: team, physician, advanced practice provider	5.5	2	No	87%
Unit of effort: clinical full-time equivalent (cFTE), total effort	6	3	No	87%
Type of work: direct care, teaching, comanagement	6	2	No	100%
Composition of teams	5.5	1	Yes	93%
Case-mix index	5.5	1	Yes	93%
Observed to expected	6	1	Yes	80%
Per work relative value unit	5	3.5	No	60%
Per case	5	3.5	No	60%

[1]Green highlighting indicates *both* consensus across the panelist (IQR <=1) *and* agreement that the metric is moderately or highly relevant (percent >= 75%).
[2]Orange highlighting indicates *either* consensus across the panelist (IQR <=1) *or* agreement that the metric is moderately or highly relevant (percent >= 75%).

© 2023 Burden M et al. *JAMA Network Open.*

eTable 2. Delphi Panel Round 3 Results

Measures	Median	IQR	Consensus	% Agreement Moderately or Very Relevant
Overall score: scoring system that includes length of stay, total visits, average dc time, patient complexity, work relative value units	6	1	Yes	100
Clinical documentation query response rates	5	1	Yes	92
Appropriate billing codes; including for prolonged visits, critical care time, counseling, coordinating care	5	1	Yes	92
Major complication or comorbidity capture rates	5	2	No	92
Diagnosis related groups	5	1	Yes	85
Turnover, intent to leave	7	1	Yes	100
Engagement	6	0.5	Yes	100
Work-life satisfaction	6	1.5	No	100
Discharges before noon	4	3	No	69
Percent early discharge	4	2	No	75
Length of stay	5	2	No	100
Bed turnover	4	2	No	92
Time to procedure: from order	3	2	No	77
Time spent on transfer center calls	4	2	No	100
Encounters: admissions, follow ups, discharges, consults	6	0	Yes	100
Encounters: critical care time, procedures	6	2	No	100
Shifts worked	6	0	Yes	100
Average starting census	6	0	Yes	100
Follow-up visit ratio: number of discharges/follow-up encounters for patients still hospitalized for a given period of time expressed as a percentage	6	1	Yes	100
Collections	4	2	No	91
Subsidy per full-time equivalent (FTE)	5	2	No	91
Professional fee charges/collections: per admission/per hospitalization	5	2	No	100
Percent of division revenue from professional billing	5	2	No	91
Percent working minutes billed per day	5	4	No	73
Financial support from hospital	5	1	Yes	100
Revenue beyond clinical work	4.5	2	No	83
Downstream clinical income from having hospitalists	5	1.5	No	92
Dollar stream from outside hospital calls	5	1	Yes	100
Cost of care	6	1	Yes	100
Savings from readmissions avoided	6	1	Yes	92

© 2023 Burden M et al. *JAMA Network Open.*

Intensive care unit days avoided	6	1	Yes	92
"Pajama Time": hours spent in the electronic health record from home or outside scheduled shift	6	1	Yes	100
Average number of interruptions experienced by a hospitalist while working directly with a patient/family	5	1	Yes	92
Rates of multitasking	6	1	Yes	92
Number of orders placed: electronically or verbally	5	1	Yes	92
Coordination time: complexity of the patient by numbers of consultants involved	6	2	No	100
Pages/calls/messages per shift: # and time	6	1	Yes	100
Time spent on discharge planning	6	1	Yes	100
Time spent in the electronic health record: including breakdown on communication in electronic health record, documentation	6	2	No	100
Time spent on clinical decision making	6	1	Yes	100
Patient acuity	6	1	Yes	100
Non-relative value unit generating work: outside hospital transfer calls, peer to peer	5	1	Yes	100
Number of interactions with other care team members	5	2	No	100
Measure of patient intensity of care: number of orders, number of procedures, geographical location	6	1	Yes	100
Hospitalist to patient ratio	6	1	Yes	100
Presentation prep and performance time	5	1	Yes	100
Self-assessment of quality of care	4	1	Yes	92
Number of additional shifts worked beyond scheduled shifts	5	1	Yes	100
Quality of communication	5	0	Yes	100
Number of consults called	5	0	Yes	100
Jeopardy or paid time off used	4	1	Yes	92
Number of hospitalists by rank	5	1	Yes	100
Number of hospitalists by position	5	0	Yes	92
Number of hospitalists by seniority	5	1	Yes	92
Number of hospitalists by teaching status	5	0	Yes	92
Number of hospitalists by research status	5	0	Yes	85
Hospitalist demographics: age, gender, years with division	5	1	Yes	100
Rates of offer acceptance	5	1	Yes	92
Medication safety: high risk meds, error rate	5	1	Yes	92
Foley management	3	1	Yes	85
Rate of readmission	5	2	No	85
Rate of inhospital mortality, observed to expected	5	2	No	92
Antibiotic usage	5	1	Yes	92
Number or rate of appropriate transfers	4	1	Yes	92

© 2023 Burden M et al. *JAMA Network Open.*

Number of patients discharged before medically ready per month	4.5	1	Yes	100
Rates of hypoglycemia	5	1	Yes	100
Rates of contradictory medical cocktails	5	1	Yes	92
Documentation of reason for dual antiplatelets or anticoagulant	5	1	Yes	92
Rates of appropriate venous thromboembolism prophylaxis	5	1	Yes	100
Participation in quality/safety committees	5	1	Yes	100
Delirium precautions initiated	5	1	Yes	92
Medication reconcilliation	5	2	No	92
Follow-up appointments made at discharge	5	1	Yes	92
Diagnostic error rate	6	1	Yes	92
Percent warm handoffs to primary care provider	5	1	Yes	100
Number of excess daily labs	5	1	Yes	92
Number of excess daily clinical procedures	5	1	Yes	92
Percent of patients with addressed goals of care	4	1	Yes	92
Percent of patients who are non-English speaking for whom interpretation is used	5	1	Yes	92
Group education initiatives: e.g., in-hospital and discharge opiate utilization	5	1.5	No	100
Best practice order set utilization	5	1	Yes	100
Goal-directed therapy utilization	4	1	Yes	92
Agency for Healthcare Research and Quality (AHRQ) Hospital Survey on Patient Safety Culture	4	2	No	85
Rapid response team or code blue calls	5	2	No	100
Intensive care unit readmission rate	5	2	No	92
Rate of unanticipated transfer from floor to intensive care unit	5	1	Yes	92
Peer feedback on quality of care	5	1	Yes	100
Discharge summary completion rate	5	1	Yes	100
Clinical documentation completeness	5	1	Yes	92
Communication and collaboration	5.5	1	Yes	100
Meeting attendance	5	1	Yes	92
Overuse of diagnostic tests or procedures	5	2	No	92
Quality of relationships with peers and trainees	5	1	Yes	92
Trainee evaluations	5	1	Yes	100
Quality of handoffs or signouts	5	1	Yes	92
Responsiveness to feedback	5	0	Yes	92
How likely would you refer a family member for care by this clinician?	6	1	Yes	100
Assessment of professionalism	5	1	Yes	92
360 degree evaluations	5	0	Yes	100

© 2023 Burden M et al. *JAMA Network Open.*

Academic output	4	1	Yes	100
Presentations, publications, or committee participation	5	1	Yes	100
Participation in job-related activities	5	0	Yes	92
Meeting institutional requirements for advancement or promotion	4	1	Yes	92
Committee participation	5	1	Yes	100
Leadership roles	5	1	Yes	100
Dissemination of academic work through other venues	4	0	Yes	100
Pressure injuries	4	2	No	77
Central line-associated bloodstream infections	4	1	Yes	85
Catheter-associated urinary tract infections	4	1	Yes	85
Clinical documentation integrity	4.5	2	No	100
Hospital Consumer Assessment of Healthcare Providers and Systems measures	3	2.5	No	67
Amount of funding provided for academic pursuits	4	1	Yes	92
Grant funding	4	2	No	69
US News & World Report ranking	3	2	No	69
Doximity ranking	3	2	No	58
Volume/growth of system	4	1	Yes	92
Clostridium difficile infections	4	1	Yes	85
Centers for Medicare & Medicaid Services quality measures	5	2	No	75
Number of trainees mentored or supervised per month	5	1	Yes	100
Time spent with trainees in hours	5	1	Yes	100
Teaching time	5	1	Yes	100
Rates of promotion	4	2	No	100
Teaching evaluations	5	2	No	100
Number of abstracts accepted	4	1	Yes	100
Impact score	4	1	Yes	92
Grants submitted	3	1	Yes	85
Grants funded	4	1	Yes	77
Total grant funding	4	1	Yes	77
Medical education leadership positions	4	0	Yes	100
Hours of pre-clinical medical student teaching	4	1	Yes	92
Number of journal articles authored	3	1	Yes	100
Number of journal articles published	4	1	Yes	100
Hours of additional training or continual education per year	3	1	Yes	92
Letters of recommendation written	4	1	Yes	85

© 2023 Burden M et al. *JAMA Network Open.*

Interviews with students and residents	4	1	Yes	100
Clinical Competency Committee participation	3	1	Yes	92
Student preceptor hours	4	1	Yes	100
Professional organization involvement	4	0	Yes	100
Publication reviewer work	3	1	Yes	100
Hours spent in educational activities or preparation	4	1	Yes	92
Amount of teaching service by gender, race, ethnicity, language abilities	5	1.5	No	100
Academic rank and promotion by gender, race, ethnicity, language abilities	5	1	Yes	100
Leadership positions by gender, race, ethnicity, language abilities	5	1.5	No	92
Hiring by gender, race, ethnicity, language abilities	5	1	Yes	100
Applicant interviews by gender, race, ethnicity, language abilities	4.5	1	Yes	100
Percent of patients with limited English proficiency getting interpreter services	5	1	Yes	92
Pay parity by gender, race, ethnicity	6	1.5	No	100
Full-time equivalent (FTE) apportioning for non clinical roles by gender, race, ethnicity	5	1.5	No	92
Concordance of race/ethnicity of hospitalist group with patients receiving care	4	1	Yes	92
Perceptions of diversity, equity, and inclusion across division	5	1	Yes	100
Completion of institutional diversity, equity, inclusion (DEI) training	5	1	Yes	92
Participation in diversity, equity, and inclusion work	4	1	Yes	100
Use of stigmatizing language in the medical record	4	1	Yes	100
Geographic spread	5	1	Yes	100
With or without advanced practice provider	5.5	1.5	No	100
Unit of time: per shift, per day, per year	6	1	Yes	100
Unit of provider: team, physician, advanced practice provider	6	1	Yes	100
Unit of effort: clinical full-time equivalent (cFTE), total effort	6	0	Yes	100
Type of work: direct care, teaching, comanagement	6	1	Yes	100
Per work relative value unit	6	1	Yes	100
Per case	5	1	Yes	100

[1]Green highlighting indicates *both* consensus across the panelist (IQR <=1) *and* agreement that the metric is moderately or highly relevant (percent >= 75%).
[2]Orange highlighting indicates *either* consensus across the panelist (IQR <=1) *or* agreement that the metric is moderately or highly relevant (percent >= 75%).

© 2023 Burden M et al. *JAMA Network Open.*
