# Supplementary material for: Salient Measures of Hospitalist Workload
Source: JAMA Netw Open. 2023 Aug 10;6(8):e2328165. doi: 10.1001/jamanetworkopen.2023.28165 (PMC10415953; doi:10.1001/jamanetworkopen.2023.28165)
Supplement: Supplement 2. — Data Sharing Statement [file jamanetwopen-e2328165-s002.pdf]

Data Sharing Statement

Burden. Salient Measures of Hospitalist Workload. *JAMA Netw Open*. Published August 10, 2023. doi:10.1001/jamanetworkopen.2023.28165

Data
Data available: Yes
Data types: Deidentified participant data
How to access data: We can provide this data upon request, de-identified. We will make the de-identified data and associated documentation available under a data-sharing agreement that provides for: (1) a commitment to not attempting to re-identify any individual participant; (2) a commitment to securing the data using appropriate computer technology; and (3) a commitment to destroying or returning the data after analyses are completed. For each dataset produced for this project, a data dictionary will be created describing the data source, survey response rate, definitions of variables included in the dataset(s), and blank survey forms as references. Data will be delivered as CSV files to users with a data-sharing agreement. Interested parties may reach out to marisha.burden@cuanschutz.edu
When available: With publication

Supporting Documents
Document types: None

Additional Information
Who can access the data: We can provide this data upon request, de-identified. We will make the de-identified data and associated documentation available under a data-sharing agreement that provides for: (1) a commitment to not attempting to re-identify any individual participant; (2) a commitment to securing the data using appropriate computer technology; and (3) a commitment to destroying or returning the data after analyses are completed. For each dataset produced for this project, a data dictionary will be created describing the data source, survey response rate, definitions of variables included in the dataset(s), and blank survey forms as references. Data will be delivered as CSV files to users with a data-sharing agreement.
Types of analyses: As stated above
Mechanisms of data availability: As stated above.
Any additional restrictions: Please see above.
